# Exploring Emotional Dysregulation and Avoidance with Caregivers as the Mechanisms Linking Social Communication Understanding and Aggressive Behaviours

**DOI:** 10.1007/s10803-024-06276-8

**Published:** 2024-05-08

**Authors:** Emma Louise Thompson, Karri Gillespie-Smith, Ally Pax Arcari Mair, Ingrid Obsuth

**Affiliations:** 1 School of Health and Social Science, University of Edinburgh, Edinburgh, EH8 9AG UK; 2 Department of Psychological Service & Research, NHS Dumfries & Galloway, Dumfries, DG1 4AP UK

**Keywords:** Autism, Adolescents, Aggressive behaviours, Emotional dysregulation

## Abstract

Many autistic adolescents and young adults present with aggressive behaviours, which can be challenging for caregivers. The present study aimed to explore the underlying mechanisms between social communication understanding and aggressive behaviours in autistic and non-autistic adolescents, specifically the role of emotional dysregulation and its impact on avoidance with caregivers. Caregivers of autistic (n = 275) and non-autistic adolescents (n = 123) completed standardised caregiver-report questionnaires measuring social communication understanding, emotional dysregulation, avoidance between the adolescent and caregiver and aggressive behaviours. A serial mediation analysis indicated that levels of social communication understanding were indirectly associated with aggressive behaviours. This occurred through increased emotional dysregulation, which may have led to increased avoidance between the autistic and non-autistic adolescents and their caregivers. These findings support a sequential process by which adolescents with low social communication understanding are more likely to behave aggressively through being emotionally dysregulated and the impact of this on the increased avoidance within the caregiver–adolescent dyad. This process was found within autistic and non-autistic adolescents, suggesting a mechanism across individuals with aggression. These findings indicate that interventions based on improving emotion regulation ability and responses between adolescents and their caregivers may aid in reducing aggressive behaviours in adolescents and young adults with lower social communication understanding.

## Introduction

Aggressive behaviours, such as hitting, biting or kicking another person or object, and self-injurious behaviours, are a common difficulty for autistic adolescents and young adults (Kanne & Mazurek, 2011). The presence of these behaviours is often associated with substantial long-term difficulties, such as these individuals experiencing restrictions to school education, reduced opportunities for interpersonal relationships, and their caregivers presenting with stress, feelings of social isolation and stigmatization (Baker et al., 2002; Hodgetts et al., 2013). While the functions of these behaviours have been previously explored (Emerson & Bromley, 1995; Fitzpatrick et al., 2016), there have been very few large-scale studies investigating the underlying mechanisms of these behaviours (e.g., Mazurek et al., 2013). Recently, researchers have begun to find evidence for a range of factors which may independently contribute to aggressive behaviours in autistic adolescents, such as emotion regulation difficulties (Cai et al., 2018; Samson et al., 2015a), and avoidance between the adolescent and their caregiver (Brown et al., 2018).

### Emotional Dysregulation

Within non-autistic adolescents, increased aggressive behaviours have been linked to difficulties with regulating emotions (Röll et al., 2012). Emotion regulation is the ability to modify one’s emotional response, including utilising appropriate strategies to alter the intensity, duration, and trajectory of both positive and negative emotions (Gross, 2002). Emotional dysregulation, on the other hand, is the inability to modify one’s emotional state in a flexible and socially appropriate manner to their current cultural context(s) (Samson et al., 2012; Waters et al., 2010). Aggressive behaviours in adolescents and young adults have been proposed to be related to the inability to regulate emotions effectively (García-Sancho et al., 2017), resulting in impulsive negative reactions (Kaartinen et al., 2014) without an apparent goal (Blair, 2016).

Within non-autistic adolescents, emotion regulation ability has been found to develop with age, with internal adaptive regulatory strategies increasing throughout adolescence and the use of maladaptive strategies decreasing (Gullone et al., 2010; Zimmermann & Iwanski, 2014). Notably, differences in emotion regulation ability, for example using fewer and less flexible strategies (Cai et al., 2018; Khor et al., 2014), are more prevalent in autistic adolescents and often result in greater and more frequent intense emotions (e.g. Joshi et al., 2018; Samson et al., 2014). As a result of difficulty with emotion regulation, autistic individuals may move towards externalising these intense emotions which can lead to more aggressive behaviours (Bos et al., 2018), particularly in social situations (Laurent & Rubin, 2004). Indeed, emotional dysregulation has been found to be associated with externalising behaviours in autistic youths, including aggression (Patel et al., 2017; Ting & Weiss, 2017). However, these studies included small sample sizes, or used single case study designs, and therefore further empirical, higher-powered investigations are required to replicate these associations within more diverse groups of autistic adolescents to produce more generalisable findings.

### Avoidance in the Caregiver–Adolescent Dyad

Recently, research is emerging regarding the interaction between caregivers and their adolescents during periods of emotional dysregulation, and the impact of this on caregivers’ ability to support them during this time (Paley & Hajal, 2022). How a caregiver interacts with an adolescent during times of heightened reactivity has been found to be associated with the escalation of externalising behaviours, such as aggression, in adolescents (e.g. Agazzi et al., 2013; Ting & Weiss, 2017). While some caregivers may act as co-regulation agents for the adolescent’s emotions, offering support and comfort, others may not be able to provide such support, possibly due to their own stress or difficulties with social communication or higher order cognition (Kopp, 1982) or the adolescent may avoid receiving support from the caregiver during this time (Banárová et al., 2022). Instead, the caregiver and the adolescent may utilise cognitive and/or emotionally avoidant coping strategies towards each other (Baumrind, 1967). This may be particularly true for autistic adolescents, who commonly experience difficulties leading to heightened emotional reactivity (e.g. Mazefsky et al., 2018a), and higher levels of aggressive behaviours (Kawabata et al., 2011).

Similarly, higher levels of positive interactions with children and adolescents, and lower levels of negative responses from caregivers, have been associated with lower levels of externalising behaviours, including aggression, in non-autistic children and adolescents (Maljaars et al., 2014). This association is a particular concern during adolescence as caregivers are required to assist with the adolescent’s need for greater autonomy whilst simultaneously being required to support the increased emotionality that can often occur during this developmental period (Van Lissa et al., 2019). A meta-analysis in non-autistic adolescents found that caregivers’ responses were associated with adolescents’ aggressive behaviours (Kawabata et al., 2011). Greater warmth and sensitivity to the adolescent’s emotions, was associated with lower levels of aggressive behaviours, while more harsh or uninvolved responses were associated with increased aggressive behaviours. Therefore, a lack of understanding, trust, communication, or emotional avoidance between the caregiver and adolescent may be a potential mechanism through which emotional dysregulation is associated with increased aggression.

### The Current Study

The evidence outlined above suggests that cognitive and emotional avoidance between caregivers and non-autistic adolescents is a mechanism that contributes to aggressive behaviours. Research to date has shown high prevalence rates of emotional dysregulation and aggressive behaviour in autistic adolescents, however, it is not known whether a lack of cognitive and emotional responses in the caregiver–adolescent dyad is impacted by the adolescent’s emotional dysregulation and in turn is related to higher levels of aggressive behaviours. Moreover, the existing studies evidencing the associations between social communication understanding, emotional dysregulation, avoidance in the caregiver–adolescent dyad and aggressive behaviours in non-autistic adolescents have used small sample sizes or case studies, which does not allow for the sequencing of these processes. The aim of the present study, therefore, was to conduct a large scale evidenced conceptualisation of these factors within autistic adolescents. This would allow investigation of how lower social communication understanding may lead to aggressive behaviours through the impact of emotional dysregulation and its impact on avoidance between adolescents and their caregivers.

However, the present study also sought to test this sequential process within non-autistic adolescents. On the one hand, autism is diagnosed based on a classification system, which proposes there are significant social communication differences in individuals with and without a diagnosis of ASD (American Psychiatric Association, 2013). On the other hand, the same clinical classification system also understands the dimensional nature to neurodevelopmental disorders, where the boundary between a disorder and neurotypical development can sometimes be unclear (American Psychiatric Association, 2022). Similarly, other researchers have proposed that autism is dimensional (Wiggins et al., 2012), and there is a continuum of social communication understanding within the general population (Grove et al., 2014, 2015). This would suggest the same associations may occur within both autistic and non-autistic adolescents, differing on level of social communication understanding rather than on diagnosis. Testing the proposed sequential process within both these samples would therefore aid in determining whether the associations found are specific to individuals with a diagnosis of autism or occur regardless of a diagnosis. It was predicted that, due to the higher prevalence rates of the measured variables within autistic adolescents (Farmer & Aman, 2011; Joshi et al., 2018; Rutgers et al., 2004), base rates for each variable would be lower in non-autistic adolescents, however the same hypothesized mechanisms, emotional dysregulation impacting on avoidance between adolescents and their caregivers, would be found.

## Method

### Participants

The study comprised a cross-sectional, quantitative, within-groups design with caregiver perspectives of two participant groups: one involving autistic adolescents, who had previously or were in the process of being diagnosed with ASD; and the other involving adolescents with no history of neurological or psychological conditions, referred to as non-autistic.

Inclusion criteria for caregivers to complete the study required them to live in the same household as the adolescent, to speak fluent English, and not to have a neurological disorder or diagnosis of an intellectual disability. For caregivers of non-autistic adolescents, the adolescent they care for was required to be aged between 11 and 21 years old and not have a history of a neurological or psychological condition. For caregivers of autistic adolescents, the adolescent they care for was required to be aged between 11 and 21 years old and have received or be in the process of receiving a diagnosis of ASD. Selection of this age range was largely based on the definition of adolescence as 10 to 19 years old (World Health Organization, 1993). However, other researchers have proposed adolescence continues until the age of 21 years (Ballarotto et al., 2018), at which point over half the adolescents within studies have moved out of their caregiver’s home (Allen et al., 2018). Since the present study was interested in the adolescent’s interactions with their caregiver, a cut off age of 21 years was selected to be as inclusive as possible whilst ensuring caregivers were able to accurately report on their interactions with the adolescent, and was in accordance with similar studies (e.g. Orsmond & Kuo, 2011). As a result, our study includes adolescents and young adults as defined by the World Health Organization (1993); however for brevity they are referred to as adolescents. Screening for these criteria occurred via a questionnaire and therefore relied on self-report.

In total, 398 caregivers were recruited: 123 caregivers of non-autistic adolescents and 275 caregivers of autistic adolescents. Demographic characteristics for both caregivers and adolescents are outlined in Table 1. Within both groups, the majority of caregivers were females, mothers of the adolescent, had lived with the adolescent all of their lives and the most common household income range (informing Social Economic Status; SES) was £25,000–£50,000. The mean age of caregivers in the non-autistic group was 44.32 years (SD = 6.53), while in the autistic group this was 45.08 years (SD = 7.78). The most common highest qualification for caregivers in the non-autistic group was a postgraduate degree, while the most common highest qualification for caregivers in the autistic group was an undergraduate degree.

**Table 1:** Demographic characteristics for all caregivers, autistic and non-autistic adolescents

	Autistic		Non-Autistic	
	N	Percent	N	Percent
**Caregiver gender**				
Male	11	4.0	10	8.1
Female	262	95.3	112	91.1
Non-binary	2	0.7	1	0.8
**Caregiver age (years)**				
20–29	3	1.1	1	0.8
30–39	68	24.7	25	20.3
40–49	123	44.7	67	54.5
50–59	74	26.9	30	24.4
60–69	6	2.2	0	0.0
70–79	1	0.4	0	0.0
**Relation to adolescent**				
Father	9	3.3	9	7.3
Mother	259	94.2	107	87.0
Carer	4	1.5	4	3.3
Stepmother	0	0.0	2	1.6
Stepfather	0	0.0	1	0.8
Grandmother	3	1.1	0	0.0
**SES**				
Less than £10,000	15	5.5	1	0.8
£10,000–£25,000	43	15.6	15	12.2
£25,000–£50,000	64	23.3	32	26.0
£50,000–£75,000	47	17.1	30	24.4
£75,000–£100,000	32	11.6	13	10.6
£100,000 +	34	12.4	16	13.0
Prefer not to say	40	14.5	16	13.0
**Caregiver’s highest qualification**				
No qualifications	8	2.9	4	3.3
GCSEs (or equivalent)	23	8.4	4	3.3
AS Levels (or equivalent)	8	2.9	2	1.6
A Levels (or equivalent)	25	9.1	5	4.1
Vocational qualification	20	7.3	14	11.4
Foundation degree	20	7.3	11	8.9
Undergraduate degree	90	32.7	32	26.0
Postgraduate degree	68	24.7	39	31.7
Doctoral degree	10	3.6	10	8.1
Other	3	1.1	2	1.6
**Time lived with adolescent**				
More than 1 year	11	4.0	18	14.6
All of their life	265	96.0	105	85.4
**Adolescent gender**				
Male	195	70.9	67	54.5
Female	67	24.4	54	43.9
Non-binary	10	3.6	2	1.6
Agender	3	1.1	0	0.0
**Adolescent gender same as at birth**				
Yes	259	94.2	119	96.7
No	16	5.8	4	3.3
**Adolescent age (years)**				
11	38	13.8	18	14.6
12	44	16.0	17	13.8
13	32	11.6	20	16.3
14	29	10.5	16	13.0
15	29	10.5	18	14.6
16	36	13.1	14	11.4
17	30	10.9	9	7.3
18	13	4.7	7	5.7
19	15	5.5	1	0.8
20	3	2.1	1	0.8
21	6	2.2	2	1.6
**Adolescent ethnicity**				
White	244	88.7	115	93.5
African/Caribbean/Black	3	1.1	2	1.6
Asian	6	2.2	2	1.6
Other	22	8.0	4	3.3
**Country where Adolescent lives**				
Scotland	34	12.4	30	24.4
England/Wales/Ireland	106	38.5	73	59.3
Other	135	49.1	20	16.3
**Adolescent’s highest qualification**				
None	7	2.5	0	0.0
Home Schooled	6	2.2	0	0.0
Still in Secondary School	209	76.0	96	78.0
GSCEs (or equivalent)	30	10.9	11	8.9
AS levels (or equivalent)	7	2.5	2	1.6
A levels (or equivalent)	4	1.5	8	6.5
Vocational qualification	6	2.2	0	0.0
Foundation degree	1	0.4	0	0.0
Undergraduate degree	1	0.4	2	1.6
Other	4	1.5	4	3.3

During data collection, an additional question asking whether the caregiver had a diagnosis of ASD was also included, based on feedback from participants. Of the 77 caregivers of non-autistic adolescents who provided data on this variable, two reported having a diagnosis of ASD themselves (1.6%). Of the 236 caregivers of autistic adolescents who provided data on this variable, 29 (10.5%) indicated they had received a diagnosis of ASD, while four (1.45%), indicated they would prefer not to say.

Within both groups, most adolescents identified as White and currently identified with the same gender they were assigned at birth. The majority were still in secondary school. The mean age of the non-autistic adolescents was 14.15 (SD = 2.35) and the majority of adolescents were from the UK. There was a relatively equal split of males and females in this group. The mean age of autistic adolescents was 14.52 (SD = 2.64), with a mean age of diagnosis of 8.03 years (SD = 4.40). There was a greater proportion of males than females in this group. There was also a split between adolescents from the UK and countries outside this, typically the USA, due to recruitment through SPARK Research Network based in North America.

### Ethical Approval

The study was conducted in compliance with the Declaration of Helsinki and received a favourable opinion by the University’s Section of Clinical and Health Psychology Research Ethics Committee.

### Procedures

Data collection took place from March to December 2021. Caregivers were recruited through advertising across several organisations and social media platforms, including the Parents Inclusion Network in Dumfries and Galloway, Discover Research Network at Autistica, Facebook, and internationally through SPARK Research Network. Interested participants were provided with a link and password to a secure online survey hosted by Qualtrics.

Once the interested caregiver opened the provided web link, they were presented with a participant information sheet providing details of the study and an option to give informed consent and proceed to complete the study; or decline participation. Caregivers were informed that their participation was voluntary, all responses were anonymous, and they could withdraw from the study at any point. A series of questions obtaining demographic information were completed and the measures reported in this study were completed as part of a battery of questionnaires. Finally, a debrief page outlining the aim of the study was provided. Caregivers did not receive any reimbursement for completing the study but had the opportunity to opt into a prize draw to receive one of two £50 gift vouchers.

### Measures

#### Social Communication Understanding: Social Communication Questionnaire (SCQ; Rutter et al., 2003)

The SCQ was utilised to measure the social communication range and understanding in the adolescents. It is a widely used screening tool for autism (Coonrod & Stone, 2005; Hus et al., 2011), for which caregivers answer 40 yes/no questions. For each question, the presence of social communication understanding is given a score of 0 and its absence is given a score of 1. Scores are summed, ranging from 0 to 39, with higher scores indicating lower social communication understanding. A proposed cut off score of 15 or more is used to indicate individuals who are likely to receive a diagnosis of autism. The SCQ has high reliability (α =  0.87; Rutter et al., 2003) and strong discriminative validity between autistic and non-autistic individuals (sensitivity = 0.88, specificity = 0.72; Chandler et al., 2007). Cronbach’s alpha in the present study was 0.87.

While there are several measures of social communication understanding, the only freely available measure, the Autism Spectrum Quotient (AQ; Baron-Cohen et al., 2006), has been found to have poor reliability (Taylor et al., 2020). The SCQ was selected over the Social Responsiveness Scale Second Edition (SRS-2; Constantino & Gruber, 2012), an alternative copyrighted measure, as it has been found to have greater sensitivity and specificity regarding autistic traits of social communication understanding (Charman et al., 2007; Moody et al., 2017). Hence, copyright for the use of 400 copies of the SCQ was obtained at the cost of £824.

#### Emotional Dysregulation: The Emotion Dysregulation Inventory (EDI; Mazefsky et al., 2018)

The EDI was used to measure the degree of the adolescent’s emotional dysregulation. It is a caregiver-report questionnaire, measuring emotional dysregulation in autistic children and adolescents. It comprises 30 items rated on a five-point Likert scale, ranging from 0 (Not at all) to 4 (Very severe). Twenty-four items measure emotional reactivity and difficulty down-regulating negative emotions while the remaining six items measure dysphoria. In the present study, only the emotional reactivity subscale was used as scores cannot be combined across scales. Scores for this factor were summed, ranging from 0 to 96, with higher scores indicating greater emotional dysregulation. Within autistic and non-autistic samples, the EDI has excellent internal reliability (α = 0.97) and validity (α = 0.94; Mazefsky et al., 2018, 2020). Cronbach’s alpha in the present study was 0.98.

#### Avoidance in the Caregiver–Adolescent Dyad: The Revised Inventory of Parent Attachment (R-IPA; Johnson et al., 2003)

The R-IPA was used to measure the cognitive and emotional responses between the caregiver and the adolescent. It is a revised version of The Inventory of Parent and Peer Attachment (IPPA; Armsden & Greenberg, 1987), measuring a caregiver’s perception of the quality of their interactions with their child. It comprises 30 items rated on a five-point Likert scale, ranging from 1 (Almost never or never true) to 5 (Almost always or always true), with 14 items reverse coded. Items are split into two factors: one measuring trust and avoidance and the other communication. A 22-item version of the questionnaire was used, reflecting the final inventory produced by the authors, based on the removal of 8 items with an inadequate factor loading onto the two subscales (Johnson et al., 2003). Within this, only questions loading onto the trust/avoidance subscale were used, for example “I don’t like being around my adolescent” and “My adolescent trusts my judgment” (see Online Resource 1 for the full questionnaire), with lack of trust indicating an emotional avoidance between the adolescent and the caregiver. Scores for these items were summed, ranging from 22 to 110 with a higher score indicating lower avoidance. These were then reversed scored to aid interpretation. The trust/avoidance subscale of the R-IPA has been found to have high reliability (α = 0.91, Johnson et al., 2003) and convergent validity, correlating negatively with other related variables, such as interpersonal relations (*r* = − .32; Johnson et al., 2003). Cronbach’s alpha in the present study was 0.93.

#### Aggressive Behaviours: Children’s Scale for Hostility and Aggression: Reactive/Proactive (C-SHARP; Farmer et al., 2016; Farmer & Aman, 2011)

The C-SHARP was used to measure the frequency of aggressive behaviours carried out by the adolescent. It is a caregiver-report questionnaire measuring aggressive behaviours in children and adolescents with developmental disorders. It comprises 40 questions split into five subscales, measuring verbal aggression, bullying, covert aggression, hostility, and physical aggression. Each item is rated on a four-point Likert scale, ranging from 0 (Does not happen) to 3 (Severe and/or very frequent problem). Scores for each subscale are averaged, with higher scores indicating more aggressive behaviour. The C-SHARP has acceptable to excellent internal reliability within a sample of autistic children and adolescents (α = 0.77 to 0.91; Farmer et al., 2016). While validity remains undetermined, the questionnaire outcomes reflect some expected differences between children with different developmental disorders (Farmer & Aman, 2011). Cronbach’s alpha in the present study was 0.97.

### Data Analysis

Data analyses were conducted using SPSS (version 27). Of the 506 participants who began the study, 97 participants withdrew after completing the demographic information. Inspection of the data indicated that participants who withdrew from the study were significantly more distant relations to the adolescent (*t*(504) = 2.47, *p* = .014, *d* = 0.25) and had lived with the adolescent for significantly less time (*t*(504) = − 2.74, *p* = .006, *d* = 0.27) than those who completed the study. It is possible therefore that the sample who withdrew did not feel able to comment on the questions within the study and as a result did not complete them. As such, the findings of the study cannot be generalised beyond caregivers who are close relatives to the adolescent and have lived with the adolescent for a considerable time.

The quality of the data of the remaining 409 participants were inspected to identify any illogical responses, continuous repeated responses on questionnaires or insufficient time to be able to appropriately complete the questionnaires. For example, if the demographic information was deemed inaccurate, such as reporting the adolescent had received their autism diagnosis at an older age than they were reported, had a higher number of older siblings than their total number of siblings, or if participants selected the same answer for each question across multiple questionnaires, these participants were removed. As a result, the data from eight participants were excluded. Comparison of this data indicated that excluded caregivers (Mean = 37.3, SD = 7.59) were significantly younger than those who were included (Mean = 44.9, SD = 7.45; *t*(407) = − 2.86, *p* = .004, *d* = 1.01), which may reflect several younger adults completing the study for entry into the prize draw rather than completing the study based on valid data.

Further inspection of the data from 401 participants indicated that 0.35% of the data was missing from 78 participants. Little MCAR test indicated the data were not missing at random, *X*^2^(14231) = 15105.65, *p* < .001. Hot Desk Imputation (Myers, 2011) was used by replacing missing values with the data of a similar participant that matched the adolescent’s age, gender and autism status (see Andridge & Little, 2010; Sande, 1983 for more information). The data could not be imputed for three participants, and these were excluded from further analyses. This resulted in a total sample size of 398, with 275 within the autistic group and 123 participants within the non-autistic group. A sensitivity analysis was conducted to determine whether the imputation of these missing values impacted on the findings. The data was reanalysed based on listwise deletion and no significant differences in the most complex regression analyses were found.

Pearson’s correlation coefficients were then inspected to assess the associations between the predictor and outcome variables based on Cohen’s (1992) criteria for small (0.10), medium (0.30) and large (0.5) associations. These findings were used to verify the suitability of the intended conditional processes analyses.

A serial mediation analysis was planned to examine whether emotional dysregulation and avoidance with caregiver mediate the association between social communication understanding and aggressive behaviours. A serial mediation analysis assumes both mediators are correlated with each other and allows for controlling for the separate influence of each mediator while determining the influence of both mediators on the association between the predictor and outcome variable. Using model 6 of the SPSS macro PROCESS (Version 4; Hayes, 2018), social communication understanding was entered as the predictor variable, aggressive behaviours as the outcome variable, and emotion dysregulation and avoidance with caregiver were entered as mediators. Independent models were run separately for the two participant groups. Path coefficients were reported in completely standardised forms (Hayes, 2018). Bootstrapping was used to overcome the potential influence of the skewed data, with sampling repeated 10,000 times, as recommended by Hayes (2018). To determine the significance of any effects, 95% confidence intervals were used.

## Results

### Group Differences

Descriptive statistics were computed for all study variables, see Table 1 for demographic variables and Table 2 for outcome variables. Independent samples t-tests were conducted to determine whether there were any significant differences in demographic information between autistic and non-autistic adolescents, specifically age, gender and SES. No significant differences were found.

**Table 2:** Predictor and outcome variables for both autistic and non-autistic adolescents

	Autistic		Non-autistic		Comparison		
	Mean	SD	Mean	SD	*t*	*p*	*d*
SCQ	18.8	6.74	12.0	7.03	9.20	< .001	.099
EDI	43.5	23.3	34.7	25.3	3.39	< .001	0.36
R-IPA	56.6	11.1	54.0	12.7	2.05	.041	0.22
C-SHARP	38.8	29.4	31.9	29.1	2.18	.030	0.24

*SD* standard deviation, *SCQ* Social Communication Questionnaire, *EDI* Emotion Dysregulation Inventory, *R-IPA* Revised Inventory of Parent Attachment, *C-SHARP* Children’s Scale for Hostility and Aggression: Reactive/Proactive

Independent samples t-tests were also conducted on the predictor and outcome variables between the two groups. As predicted, autistic adolescents had lower social communication understanding and significantly more emotional dysregulation, avoidance with caregiver, and aggressive behaviours than non-autistic adolescents.

### Correlations

Bivariate Pearson’s correlations were conducted between the demographic, predictor, and outcome variables for each group to identify any associations (see Table 3).

**Table 3:** Correlations between demographic, predictor and outcome variables

Variable	1.	2.	3.	4.	5.	6.	7.
Autistic adolescents							
1. SES	–						
2. Adolescent age	− 0.05	–					
3. Adolescent gender	− 0.03	0.00	–				
4. SCQ	− 0.19**	0.05	0.07	–			
5. EDI	− 0.31**	0.00	0.15*	0.36**	–		
6. R-IPA	− 0.14*	0.07	0.14*	0.33**	0.64**	–	
7. C-SHARP	− 0.21**	0.04	0.10	0.22**	0.74**	0.66**	–
Non-autistic adolescents							
1. SES	–						
2. Adolescent age	− 0.03	–					
3. Adolescent gender	0.05	− 0.01	–				
4. SCQ	− 0.23*	0.12	− 0.10	–			
5. EDI	− 0.17	− 0.03	0.09	0.70**	–		
6. R-IPA	0.00	0.05	0.14	0.58**	0.77**	–	
7. C-SHARP	− 0.12	− 0.24**	− 0.02	0.53**	0.76**	0.70**	–

*SES* Social Economic Status, *SCQ* Social Communication Questionnaire, *EDI* Emotion Dysregulation Inventory, *R-IPA* Revised Inventory of Parent Attachment, *C-SHARP* Children’s Scale for Hostility and Aggression

*p < .05, **p < .01

#### Autistic Adolescents

For the autistic adolescents, adolescent gender was associated with emotional dysregulation and avoidance with caregiver: female identity was correlated with more emotion dysregulation, while male identity was correlated with more avoidance with caregiver. Lower SES was associated with lower social communication understanding and more emotional dysregulation, avoidance with caregiver and aggressive behaviours. Although SES may therefore have had an impact on aggressive behaviours in this group, since demographic variables did not differ between groups, and to keep analyses consistent across groups, the analysis was run without controlling for SES. However, afterwards a sensitivity analysis was conducted, including SES as a covariate, to determine whether SES had impacted on any of the analyses, of which no effect was found.

All outcome variables were correlated. Low social communication understanding was positively correlated with emotional dysregulation, avoidance with caregiver, and aggressive behaviours.

#### Non-autistic Adolescents

Between the demographic variables and outcome variables, lower SES was associated with lower social communication understanding. More aggressive behaviours was also associated with younger adolescents.

All outcome variables were significantly correlated. Social communication understanding was positively correlated with emotional dysregulation, avoidance with caregiver and aggressive behaviours.

### Serial Mediation Analyses

In order to test the hypothesis, a serial mediation analysis was conducted to determine whether **e**motional dysregulation and avoidance with caregiver are the sequential processes through which low social communication understanding is linked to aggressive behaviours.

#### Autistic Adolescents

**Fig. 1:**
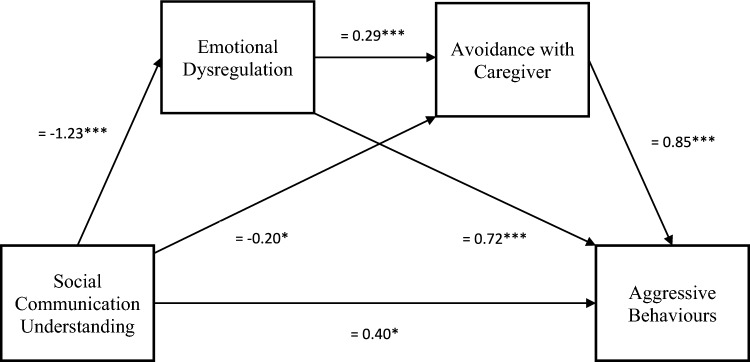
Direct and indirect effects of the serial mediation of social communication understanding predicting aggressive behaviours for autistic adolescents. *Note* All coefficients are standardized, *p $\le$ .05, **p $\le$ .01, ***p $\le$ .001

The overall regression model predicting aggressive behaviours from social communication understanding, emotional dysregulation and avoidance with caregiver was significant, explaining 61.1% of the variance in aggressive behaviours (*R*^2^ = .611, *F*(3, 271) = 142.1, *p* < .001), as shown in Fig. 1. Consistent with the hypothesis, the serial mediation effect from low social communication understanding to increased aggressive behaviours through increased emotional dysregulation and increased avoidance with caregiver was significant (*β* = 0.30, SE = 0.81, 95% CI 0.16, 0.47). Lower social communication understanding also predicted more avoidance with caregiver and more emotional dysregulation predicted more aggressive behaviours. However, there was also a significant direct effect of low social communication understanding on aggressive behaviours (*β* = − 0.39, *SE* = 0.18, *p* = .028), indicating a partial mediation. To determine whether multicollinearity was present between any variables in the analysis, regression analyses were run. All variance inflation factors (VIF) were relatively low (< 5).

#### Non-autistic Adolescents

**Fig. 2:**
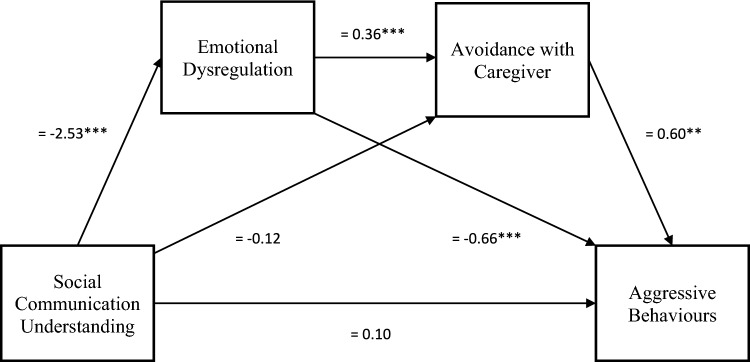
Direct and indirect effects of the serial mediation of social communication understanding predicting aggressive behaviours for non-autistic adolescents. *Note* All coefficients are standardized, *p $\le$ .05, **p $\le$ .01, ***p $\le$ .001

The overall regression model predicting aggressive behaviours from social communication understanding, emotional dysregulation and avoidance with caregiver was significant, explaining 60.5% of the variance in aggressive behaviours (*R*^2^ = .605, *F*(3, 119) = 60.8, *p* < .001), as shown in Fig. 2. Consistent with the hypothesis, the serial mediation effect from lower social communication understanding to increased aggressive behaviours through increased emotional dysregulation and increased avoidance with caregiver was significant (*β* = 0.55, SE = 0.23, 95% CI = 0.14, 1.04). Moreover, the direct effect of social communication understanding to aggressive behaviours was not significant (*β* = − 0.10, *SE* = 0.34, *p* = .771). The presence of an indirect effect in the absence of a direct effect indicates a full mediation (Baron & Kenny, 1986). However, social communication understanding did not directly predict avoidance with caregiver and emotional dysregulation directly predicted less aggressive behaviours.

### Additional Exploratory Analyses

Additional analyses were conducted on the serial mediation to determine the possible bidirectional nature of the effects found. The regression models were rerun investigating the impact of social communication understanding on aggressive behaviours through avoidance with caregiver and emotional dysregulation. Within both autistic and non-autistic adolescents, the serial mediation effects were significant and avoidance with caregiver directly predicted emotion dysregulation.

A second serial mediation was also run to determine whether **e**motional dysregulation and aggressive behaviours are linked to increased avoidance with caregivers. Within both autistic and non-autistic adolescents, the overall regression models were significant. The serial mediation effects from lower social communication understanding to increased avoidance with caregiver through increased emotional dysregulation and increased aggressive behaviours were significant. As a result, the findings related to the predicted links will be interpreted with these results in mind.

## Discussion

The aim of the present study was to test a conceptual model of the factors related to the association between social communication understanding and aggressive behaviours in autistic and non-autistic adolescents. Specifically, the study aimed to evidence the sequential process by which adolescents and young adults with low levels of social communication understanding are more likely to behave aggressively through being emotionally dysregulated and the impact of this on the amount of cognitive and emotional avoidance between them and their caregiver.

Within the present study, autistic adolescents had significantly lower rates of social communication understanding and higher rates of emotional dysregulation, avoidance with caregiver, and aggressive behaviours compared to non-autistic adolescents, as predicted. These findings highlight the prevalence of these factors in relation to autistic adolescents, and are in accordance with previous research (Brown et al., 2018; Cai et al., 2018; Mazurek et al., 2013; Ozsivadjian et al., 2021; Samson et al., 2015a).

The present study also found evidence to support the proposed sequential process between social communication understanding and aggressive behaviours. For autistic adolescents, lower social communication understanding led to more aggressive behaviours through the mechanisms of greater emotional dysregulation impacting on higher cognitive and emotional avoidance with caregivers. These findings build upon research indicating that within adolescents, lower levels of social communication understanding are associated with greater emotional dysregulation (Cai et al., 2018; Samson et al., 2014), which is related to avoidant cognitive and emotional responses in the caregiver–adolescent dyad (Paley & Hajal, 2022), which is related to increases in adolescents’ aggressive behaviours (e.g. Armstrong et al., 2014; Lucyshyn et al., 2007).

However, the additional exploratory analyses found that the reverse sequence was also significant: for both autistic and non-autistic adolescents, lower social communication understanding led to more avoidance between the adolescent and caregiver, impacting on higher emotional dysregulation, which led to more aggressive behaviour. Previous research has found emotional closeness moderated the relationship between adolescents’ attachment avoidance levels and their emotion regulation strategies (Costa et al., 2022), while a recent meta-analysis found a small significant negative association between a warm and supportive caregiver–adolescent dyad and emotion dysregulation (Goagoses et al., 2023). Therefore, more sensitive and responsive interactions between the adolescent and their caregivers are likely to help adolescents regulate their feelings of distress, leading to less emotion dysregulation (Stern, 2005).

Similarly, the present study also found lower social communication understanding led to increased avoidance in the caregiver–adolescent dyad through increased emotional dysregulation and increased aggressive behaviours. While the majority of previous research has investigated the impact of caregiver–adolescent interactions and caregiver burnout on aggressive behaviour (e.g. Yuan et al., 2022), the present study indicates that adolescent aggressive behaviours are likely to also impact on the interactions between the adolescent and the caregiver, through possible strain in the dynamics or caregiver burn out. Further research exploring the mechanism of the effect in this direction is required, therefore, to help understand the impact of this further.

In autistic adolescents, after the impact of emotion dysregulation and avoidance between the adolescent and caregiver had been removed from the analysis, increased levels of social communication understanding continued to be directly associated with more aggressive behaviours. This indicates that the process of being emotionally dysregulated and in turn experiencing avoidant cognitive and emotional responses with the caregiver does not fully explain the effect of social communication abilities on aggressive behaviours. Moreover, despite the overall positive association between low levels of social communication understanding and aggressive behaviours, when removing the variance explained by the indirect process, this direct effect was negative. One possibility for this difference could reflect suppression within the analysis, in which multicollinearity between variables may have led to an unreliable and unstable estimate of the regression coefficient (MacKinnon et al., 2000). However, this analysis indicated that although there was some dependence between variables, this was insufficient to warrant a significant multicollinearity concern (James et al., 2013). Instead, another possibility for these findings may reflect the different components of autism measured by the SCQ, which may have differential effects on aggressive behaviours. For example, one question asks whether the adolescent has any ways of moving their hands or fingers, such as flapping or moving their fingers in front of their eyes; stimming behaviours (i.e. self-stimulatory behaviours) such as these may be beneficial for autistic adolescents in times of heightened emotion. For example, Kapp et al. (2019) found that autistic adults report stimming is an adaptive mechanism that helps them to soothe or communicate intense emotions. Therefore, when the emotional dysregulation and avoidance in the caregiver–adolescent dyad mechanism is taken into account, the remaining small effect of the lower social communication understanding may negatively impact on aggressive behaviours.

However, within non-autistic adolescents, the effect of low social communication understanding on aggressive behaviours was fully explained by the process of being emotionally dysregulated impacting on increased cognitive and emotional avoidance with the caregiver. Together, these findings indicate that the same mechanisms between social communication understanding and aggressive behaviours exist across the diagnostic category of ASD, supporting a more dimensional approach to autism (Wiggins et al., 2012). However, it is possible the functions of the aggressive behaviours may differ between autistic and non-autistic adolescents, which was not measured in the present study, for example releasing stress (Bronsard et al., 2010) or sensory overstimulation (van den Boogert et al., 2021). Future research exploring of the function of these behaviours would enable a more enriched understanding ways to support adolescents and young adults during this time and determine any differences across individuals with aggression.

### Strengths and Limitations

The present study has several strengths. Firstly, it is one of a select number of large-scale studies investigating the underlying mechanisms of aggressive behaviours in autistic adolescents. Specifically, the serial nature of the mediation allowed the investigation of two processes, emotional dysregulation and avoidance with caregivers, and their sequencing in relation to aggressive behaviours, thus providing a more comprehensive possible target for interventions. However, while the findings demonstrated the impact of emotion dysregulation on avoidance between the caregiver and adolescent, evidence was also found for the reverse direction of avoidance in the caregiver–adolescent dyad on emotion dysregulation. Therefore, while there was a theoretical rationale for the direction of the mechanisms investigated in the present study, future research is required to explore alternative directions of these mechanisms to gain understanding of possible interactions with additional variables.

Furthermore, the present study used caregiver observations of heterogeneous samples: autistic and non-autistic adolescents who reported a variety of additional mental and physical conditions typical of this population, such as ADHD and anxiety (Leitner, 2014; Rodgers & Ofield, 2018). While caution should be taken in drawing conclusions specifically relating to social communication understanding, the findings of the present study are generalisable to the population typically presenting to services for aggressive behaviours, regardless of diagnoses, providing high ecological validity.

The present study also has several limitations. Firstly, while the SCQ was used to measure social communication understanding, the use of social communication questions within adaptive behaviour assessments, such as the Adaptive Behaviour Assessment System 3rd edition (ABAS-3; Harrison & Oakland, 2018), may have provided more enriched information on these abilities. Adaptive behaviour questions are designed to measure ability rather than screen the presence or absence of certain behaviours, and therefore may have allowed for further analysis of the social communication understanding of the individuals within the present study compared to the SCQ. Secondly, the measures used relied on caregiver-reports of their adolescent. Reporter characteristics, such as the caregiver’s mood, can commonly introduce bias when reporting on multiple report scales. This may have increased the risk of bias due to under or over-reporting of difficulties and may differ to what adolescents might have reported had they completed the study themselves. Caution should also be taken when considering the caregiver-reporting of the adolescent’s autism diagnosis, as allowing for anonymity prevented diagnoses being able to be verified by medical records. While this increases the chances of false positive responses, it is the typical method of collecting such data in the literature (e.g. Livingston et al., 2023). Moreover, Fombonne et al. (2022) found 98.8% of diagnoses were confirmed by electronic medical records in a sample of SPARK network participants, from which the majority of caregivers of autistic adolescents within the current study were recruited, indicating high validity of diagnoses within those who took part.

In addition, the study also only reports on outwardly observable behaviours mediated by social communication understanding, emotional regulation, and avoidance in the caregiver–adolescent dyad, and does not account for internalised, and thus not directly observable, experiences of emotional dysregulation and masked difficulties. This may be reflected in the association between adolescent gender and observable emotional dysregulation, due to masking being more common in autistic women and girls (Muggleton et al., 2019). Future research should seek information from multiple informants, including the adolescents and young adults, or combine self-report and behavioural measures of each factor to reduce the risk of bias. Finally, no information was provided on current medication use. Psychotropic medication, such as risperidone, are used to treat maladaptive behaviours, including aggression, in autistic children and adolescents (Rimmington, 2017). This may have influenced some of the associations found, as this may have also heightened neurological thresholds or influenced behavioural responses (van den Boogert et al., 2021). A lack of this data should therefore be taken into consideration when interpreting the findings of the present study.

### Theoretical and Clinical Practice Implications

The findings of the present study suggest a potential theory to understand aggressive behaviours in autistic adolescents and young adults, adding to the current literature (Hill et al., 2014). A key component in aggressive behaviour appears to relate to the regulation of emotions within the adolescent (Mazefsky et al., 2013; Samson et al., 2015a), which reportedly may lead to less supportive interactions by the caregiver. However, the additional analyses conducted in the present study also highlighted the likely bidirectional nature of the associations found. For example, it is equally possible that avoidance in the caregiver–adolescent interactions impacts the adolescent’s emotion dysregulation. Similarly, the adolescent’s aggressive behaviours are likely a mechanism through which emotional dysregulation is associated with avoidance with the caregiver. Therefore, while theoretical rationale influenced the investigated direction of the mechanisms in the present study, and evidence was found to support these mechanisms, the effects found are likely bidirectional and causality cannot be established. As a result, future research would benefit from conducting longitudinal studies to tease apart the complexity of these associations as well as including additional variables, such as caregiver burnout, to understand when and how these mechanisms occur.

The findings of the present study also have clinical implications for a large proportion of autistic adolescents and young adults for whom aggressive behaviours are a substantial concern (Hartley et al., 2008; Kanne & Mazurek, 2011; Mazurek et al., 2013). The findings indicate that high emotional dysregulation in the adolescent leads to avoidance with their caregivers, which may further exacerbate aggressive behaviours in autistic adolescents. Creating emotion regulation interventions for these individuals may aid in reducing the association between social communication understanding and aggressive behaviours (Gross & Thompson, 2007; Mazefsky et al., 2013; Weiss et al., 2014). For example, a recent within-subjects study investigating the effective of an emotion dysregulation group treatment for autistic children and adolescents and their caregivers (Shaffer et al., 2022) found an improvement in these individuals in reactivity, emotion regulation knowledge, and flexibility, immediately following and 10-weeks post-treatment, as well as a reduction in psychiatric hospitalizations.

Furthermore, given lower social communication understanding and increased cognitive and emotional avoidance with their caregiver informing aggressive behaviours, the findings of this study are suggestive of a need for tools which may increase social understanding between the autistic adolescent and the caregiver, as well as co-regulation. Similarly, the present study also highlighted the association between greater emotional dysregulation impacting on avoidance between the caregivers and adolescent, and vice versa. Increasing training of handling negative emotional expressions and learning to react more supportively, as well as an awareness in the expectation of how more negative emotional expressions may influence caregiver reactions, may aid in reducing aggressive behaviours for these adolescents (Klinger et al., 2013). Within the present study, it is possible caregivers may have previously received such support, however the length of time the adolescent had demonstrated aggressive behaviours and the level of support received by caregivers were not measured. Likewise, the measures used within the current study did not allow for determining the caregiver’s behavioural responses to the adolescent during their times of emotion dysregulation or the level of stress the caregiver is under. These are additional elements, which may shed more light on the mechanisms found, but require further exploration. Future research measuring the level of additional support received by caregivers and the impact of this on the adolescent’s emotion dysregulation and aggressive behaviours within a bi-directional model would be beneficial to provide more understanding of the possible benefit of caregiver support and its impact on the interaction between the behaviours of adolescents and their caregivers.

However, sensitivity should be taken when informing caregivers of these findings as caregivers of adolescents with developmental disabilities often experience blame or stigma (Francis, 2012), or hold themselves responsible (Moses, 2010) for their adolescent’s difficulties. Therefore, caution should be taken ensure the findings are not interpreted by caregivers in a way that adds to the guilt they may feel about what they could or should have done differently to prevent their adolescent’s difficulties (Weintraub, 2011). To minimise such potential negative effects, a positive, non-judgemental therapeutic relationship should be established followed by feedback focusing on specific behaviours that can be improved (Hardavella et al., 2017). Moreover, it is important to convey the effects found are likely to be bi-directional, with both the caregiver’s and adolescent’s responses influencing each other, as indicated by the measure used and the additional analyses run, making it difficult to determine the extent of purely caregiver driven effects.

### Conclusion

The present study found evidence to support a sequential process by which adolescents and young adults with low social communication understanding are more likely to behave aggressively through being emotionally dysregulated and the impact of this on the reduced cognitive and emotional responses between them and their caregiver. However, additional findings indicate this mechanism is likely to be bi-directional, with avoidance between the caregiver and adolescent impacting on the adolescent’s emotion dysregulation and their aggressive behaviour impacting on avoidance in the caregiver–adolescent dyad, indicating the complexity and impact of adolescent aggressive behaviour. These process were found within both autistic and non-autistic adolescents, evidencing the mechanism across individuals with aggression. These findings indicate that interventions based on improving emotion regulation ability and interactions between caregivers and their adolescents may aid in reducing aggressive behaviours in adolescents with lower social communication understanding. Future research would benefit from investigating the bi-directional nature of the processes found through a longitudinal design to establish causality.
